# Compliance with Guidelines-Recommended Processes in Pneumonia: Impact of Health Status and Initial Signs

**DOI:** 10.1371/journal.pone.0037570

**Published:** 2012-05-22

**Authors:** Rosario Menéndez, Antoni Torres, Soledad Reyes, Rafael Zalacain, Alberto Capelastegui, Olga Rajas, Luis Borderías, Juan J. Martín-Villasclaras, Salvador Bello, Inmaculada Alfageme, Felipe Rodríguez de Castro, Jordi Rello, Luis Molinos, Juan Ruiz-Manzano

**Affiliations:** 1 Servicio de Neumología, Hospital Universitario y politecnico La Fe, CIBERES, Valencia, Spain; 2 Servei de Pneumologia, Institut Clinic del Torax, IDIBAPS, Universitat de Barcelona, CIBERES, Barcelona, Spain; 3 Servicio de Neumología, Hospital de Cruces, Bilbao, Spain; 4 Servicio de Neumología, Hospital de Galdakao, Galdakao, Spain; 5 Servicio de Neumología, Hospital de la Princesa, Madrid, Spain; 6 Servicio de Neumología, Hospital San Jorge, Huesca, Spain; 7 Servicio de Neumología, Hospital Carlos Haya, Malaga, Spain; 8 Servicio de Neumología, Hospital Miguel Servet, Zaragoza, Spain; 9 Servicio de Neumología, Hospital de Valme, Sevilla, Spain; 10 Servicio de Neumología, Hospital Dr. Negrín, Las Palmas de Gran Canaria, Spain; 11 Critical Care, Hospital Vall d’Hebron, Institut de Recerca Vall d’Hebron-UAB, CIBERES, Barcelona, Spain; 12 Servicio de Neumología, Hospital Central Asturias, Oviedo, Spain; 13 Servicio de Neumología, Hospital Germans Trias i Pujol, Badalona, Spain; D’or Institute of Research and Education, Brazil

## Abstract

Initial care has been associated with improved survival of community-acquired pneumonia (CAP). We aimed to investigate patient comorbidities and health status measured by the Charlson index and clinical signs at diagnosis associated with adherence to recommended processes of care in CAP. We studied 3844 patients hospitalized with CAP. The evaluated recommendations were antibiotic adherence to Spanish guidelines, first antibiotic dose <6 hours and oxygen assessment. Antibiotic adherence was 72.6%, first dose <6 h was 73.4% and oxygen assessment was 90.2%. Antibiotic adherence was negatively associated with a high Charlson score (Odds ratio [OR], 0.91), confusion (OR, 0.66) and tachycardia ≥100 bpm (OR, 0.77). Delayed first dose was significantly lower in those with tachycardia (OR, 0.75). Initial oxygen assessment was negatively associated with fever (OR, 0.61), whereas tachypnea ≥30 (OR, 1.58), tachycardia (OR, 1.39), age >65 (OR, 1.51) and COPD (OR, 1.80) were protective factors. The combination of antibiotic adherence and timing <6 hours was negatively associated with confusion (OR, 0.69) and a high Charlson score (OR, 0.92) adjusting for severity and hospital effect, whereas age was not an independent factor. Deficient health status and confusion, rather than age, are associated with lower compliance with antibiotic therapy recommendations and timing, thus identifying a subpopulation more prone to receiving lower quality care**.**

## Introduction

Pneumonia continues to be one of the main causes of death due to infection, and is responsible for considerable use of healthcare resources and economic burden reference CAP burden [1]. Evidence-based guidelines have been developed by scientific societies to assist physicians in the management of CAP and to reduce variability in clinical care [2]. The main purpose of the guidelines is to improve the care of patients with pneumonia. The most recent studies show that implementation of the guidelines is accompanied by an increase in the percentage of processes of care and a lower inpatient hospital mortality rate, in the first 48 hours [3] and after 30 days [4]–[6].

The decision regarding antibiotic treatment, evaluation of the severity of CAP, timing of first dose and its overall management until complete resolution all play a key role in the prognosis of the disease. The processes of care most consistently included in the evaluation of quality of care in US hospitals were [7] time until first dose, antibiotic prescribed, oxygen assessment and blood cultures. Initial antibiotic treatment is key to resolution of the infection and to outcome: mortality is higher when the treatment is inappropriate. In several cohort studies, antibiotic adherence was an independent protective factor for mortality and length of stay [8]. More controversy surrounds the number of hours until first antibiotic (AB) dose. Although it has been shown that shorter times between diagnosis and initiation of treatment improves outcome [9], [10], subsequent studies have warned of increasing prescription of unnecessary antibiotics [11]. The new recommendations establish a threshold of 6 hours for the first dose.

Oxygen assessment has also been recommended in patients presenting with suspected CAP and given the availability of a noninvasive method and the importance of rapid recognition of respiratory failure, [12] this process of care is an important goal.

Non-adherence to or low compliance with processes of care is an important issue because of the impact on outcome and because of the opportunity to improve quality of care in CAP [5], [6], [13]. Moreover, compliance with guidelines is highly variable among clinicians [14] and may be due to individual inclination [15], [16], with differences among hospital type and characteristics [2]. Halm et al [17] identified factors associated with non-adherence to guidelines for the decision to hospitalize patients with CAP, but little information is available regarding factors that influence adherence to guidelines for the selection of treatment of CAP.

We hypothesized that compliance with different processes of care may differ depending on comorbid conditions of patients, such as general health status, age or initial signs. Improving our recognition of these elements may enable us to reduce behavior-dependent barriers that lower quality of care. Efforts should be aimed at identifying factors that influence adherence to guidelines relating to patient characteristics, which are more accessible than wide variability in hospital type.

The primary objective of this study was to evaluate factors linked to compliance with three processes of care in hospitalized patients with CAP: antibiotic adherence to guidelines, first antibiotic dose <6 hours and oxygen assessment. In the study, we analyzed factors associated with comorbid conditions and general health status measured by the Charlson score, initial clinical signs that are all available at initial diagnosis.

## Methods

### Design and Study Population

Between November 2005 and November 2007, we performed a multicenter observational prospective study in 13 Spanish hospitals belonging to the Spanish National Health System. The study cohort comprised non-immunosuppressed adult patients hospitalized due to CAP, who were not admitted to an ICU and were not nursing-home residents. The study was approved by the Ethics Committees and the patients provided written informed consent.

### Processes of Care for Inpatients

The following processes of care in accordance with Spanish guidelines were recorded: 1. Measurement of arterial oxygen saturation on presentation (by pulse oximetry or arterial blood gas analysis); 2. Time until first antibiotic dose (<6 hours); 3. Antibiotic adherence to Spanish guidelines [18].

The attending physician prescribed the initial empirical antibiotic therapy. Antibiotic adherence was considered to be the following: in hospitalized CAP patients, either 3^rd^-generation cephalosporin or amoxicillin–clavulanic acid combined with a macrolide, or fluoroquinolone (3^rd^ or 4^th^ generation) in single-drug therapy. All other regimens were considered non-adherent.

Processes of care were analyzed according to the following: 1. Patient characteristics (age, vaccination status, gender, comorbid condition, toxic habits) and 2. Initial signs. Comorbidities were defined as follows: chronic obstructive pulmonary disease (COPD) was based on clinical criteria and on spirometric values (if available before or after hospital admission). Cardiac disease: treatment for coronary artery disease or congestive heart failure or presence of valvular heart disease. Renal disease: preexisting renal disease with documented abnormal serum-creatinine outside the pneumonia episode. Liver: preexisting viral or toxic liver disease. CNS disorders: symptomatic acute or chronic vascular or nonvascular encephalopathy, with or without dementia. Diabetes mellitus: diagnosis of intolerance to glucose and treatment with oral antidiabetics or insulin. Neoplastic illness: any solid tumor active at the time of presentation or requiring antineoplastic treatment within the previous year. A Charlson comorbidity index was calculated [19]. Initial signs and symptoms analyzed were fever, dyspnea, acute confusion (disorientation with regard to person, place, or time that is not known to be chronic, stupor, or coma), respiratory rate (RR), heart rate (HR), systolic blood pressure <90 mmHg.

### Statistical Study

#### Univariate analysis

Statistical analyses were performed using the SAS 8.2 software program. Qualitative variables were compared using the χ^2^ test. Differences in quantitative variables were assessed using ANOVA or the Kruskal-Wallis test, where appropriate. Values of p≤0.05 were considered significant. Variables for univariate analysis were dichotomized as follows: age (≥65 years), fever (≥37.8°C), tachycardia (≥100 bpm), tachypnea (≥30 bpm), hypotension (systolic blood pressure ≤90 mmHg), and hypoxemia (PaO_2_≤60 mmHg or oxygen saturation ≤90%).

#### Multivariate analysis

Three logistic regression analyses were performed to predict compliance with each process of care: antibiotic adherence to guidelines, time to first dose <6 hours and initial oxygen assessment. For each mathematical model, independent variables were those with p<0.1 in the univariate analyses.

Two more logistic regression analyses were performed to assess independent variables associated with compliance with two or three combined processes of care: antibiotic adherence and first dose <6 hours, and those two processes plus initial oxygen assessment.

The logistic regression analyses were performed using a likelihood ratio-based stepwise method. Variables that obtained a p-value ≤0.05 were considered significant and their odds ratios with 95% confidence intervals were calculated. Colinearity amongst the independent variables was assessed by means of the Pearson’s correlation coefficient. Those variables strongly correlated were excluded from the multivariate analysis. The Hosmer and Lemeshow goodness-of-fit test was used to evaluate the adequacy of the models [20].

## Results

The original cohort comprised 4374 patients from 13 Spanish hospitals. We included 3844 patients in the study after excluding nursing home patients (n, 237) and those admitted to the ICU (306). The main demographic characteristics, comorbidities and PSI scores are shown in Table 1.

**Table 1:** Characteristics of the whole cohort (N = 3844).

Characteristic	Patient
Age	66.1±18.2
Sex (M/F)	2538 (66.0)/1306 (34.0)
Smoking	823 (21.4)
Alcohol	408 (10.6)
Pneumococcal vaccination	387 (11.7)
Influenza vaccination	1677 (50.4)
**Comorbidity**	
Diabetes	821 (21.4)
Hepatopathy	147 (3.8)
Cardiopathy	550 (14.3)
Kidney failure	250 (6.5)
Neoplasia	211 (5.5)
CNS disease	410 (10.7)
COPD	919 (24.4)
**PSI**	
I-III	2140 (55.7)
IV-V	1704 (44.3)

Data are No. (%) or mean ±SD.

Abbreviation: M/F indicates male/female; CNS, central nervous system; COPD, chronic obstructive pulmonary disease; PSI, pneumonia system index.

The overall rates for the processes of care were as follows: 3466 patients (90.2%) had oxygen assessment, 2791 (72.6%) received antibiotics in compliance with guidelines (645 patients were treated with narrower-spectrum antibiotics regimens and 408 with different combinations) and 2822 (73.4%) received the first dose of antibiotic within 6 hours of arrival at the emergency department. The combination of two processes of care was found in 2024 (52.7%) of patients and three processes of care in 1828 (47.6%) of patients.

### Univariate Analyses

Demographic characteristics, comorbid condition, initial signs and symptoms were analyzed with respect to each processes of care. The variables found to be significant are shown in Table 2.

**Table 2:** Patient characteristics depending on each process of care.

	AB Adherent SEPAR		First AB dose >6 h		Oxygen assessment	
	No/Yes	P value	No/Yes	P value	No/Yes	P value
	(n = 1039/2791)		(n = 2822/842)		(n = 378/3466)	
**Variable (%)**						
**Demographic**						
Age >65	63.3/63.0	0.8	63.2/64.0	0.7	50.9/64.4	<0.001
Male sex	67.3/65.3	0.3	66.7/64.0	0.2	63.5/66.3	0.3
Alcohol	11.9/10.2	0.031	10.1/12.1	0.3	10.1/10.7	0.9
**Comorbidities**						
COPD	25.9/23.8	0.2	24.9/23.8	0.5	12.9/25.6	<0.001
Heart disease	14.0/14.5	0.7	13.8/16.0	0.1	13.5/14.4	0.6
CNS disease	11.0/10.6	0.7	10.4/12.3	0.1	8.7/10.9	0.2
Diabetes	20.7/21.7	0.5	21.2/22.8	0.3	19.8/21.5	0.4
Charlson, mean ±SD	1.8±3.4/1.4±1.6	<0.001	1.5±2.4/1.6±1.8	0.3	1.4±5.5/1.5±1.7	0.8
Neoplasma	8.2/4.5	<0.001	5.0/6.8	0.052	4.5/5.6	0.4
**Sign and symptoms**						
Fever	79.0/77.7	0.4	78.8/76.8	0.2	84.3/77.2	0.002
Confusion	14.7/9.3	<0.001	10.4/12.6	0.066	6.7/11.2	0.007
RR >30	21.9/16.1	<0.001	17.8/17.2	0.7	14.6/18.0	0.1
HR >100	43.4/37.9	0.002	40.6/36.5	0.031	43.2/39.0	0.1
Systolic pressure <90	4.6/3.0	0.013	3.8/2.5	0.081	3.8/3.4	0.7

Data are presented as % or mean ±SD.

Abbrevations: AB, indicates antibiotic; CNS, central nervous system; COPD, chronic obstructive pulmonary disease; M/F, male/female; PSI, pneumonia system index; SEPAR, Sociedad Española de Neumología y Cirugía Torácica; HR =  heart rate; NS, not significant; RR, respiratory rate.

### Multivariate Analyses

The independent variables associated with each process of care (antibiotic adherence to guidelines, first dose >6 hours and oxygen assessment) are shown in Table 3.

**Table 3:** Multivariate logistic regression analyses for each processes of care.

	AB adherent	First AB dose >6 h	Oxygen Assessment
Variable	OR (95% CI)	OR (95% CI)	OR (95% CI)
**Demographic**			
Age >65	…	…	1.51 (1.1–2.06)**
**Comorbid condition**			
COPD	…	…	1.80 (1.14–2.80)**
Charlson (+1)	0.91 (0.86–0.95)***	…	…
**Sign and symptoms**			
Fever	…	…	0.61 (0.41–0.92)**
Confusion	0.66 (0.51–0.85)***	…	…
RR >30	…	…	1.58 (1.04–2.42)*
HR >100	0.77 (0.65–0.91)***	0.75 (0.62–0.90)***	1.39(1.01–1.91)*

Logistic regression analyses were adjusted by centre (p<0.001).

Abbreviations: CI indicates confidence interval; OR, odds ratio. AB, antibiotic; HR heart rate; RR, respiratory rate.

* p<0.05 and >0.01 **p:0.01 ***p<0.01.

In terms of comorbid conditions, a high Charlson score was associated with lower antibiotic (AB) adherence while COPD and patients over 65 years of age were associated with higher oxygen assessment. With regard to signs, patients with tachycardia received the first antibiotic dose earlier, although they received more nonadherent antibiotic treatments. Patients with confusion received also more nonadherent antibiotic treatments.

The results of the multivariate analyses to predict compliance with two or three processes of care are shown in Table 4.

**Table 4:** Multivariate logistic regression analyses for compliance with two (AB adherent to SEPAR) or three processes of care (AB adherent to SEPAR and first dose <6 h plus oxygen assessment).

	Two processes of care	Three processes of care
	AB adherent and first dose <6 h	Two + oxygen assessment
**Variable**	OR (95% CI)	OR (95% CI)
**Comorbid condition**		
Charlson (+1)	0.92 (0.88–0.96)*	
Neoplasia		0.59 (0.42–0.83)*
**Sign and symptoms**		
Confusion	0.69 (0.54–0.88)*	0.71 (0.56–0.91)**

Logistic regression analyses were adjusted by hospital (p<0.001).

Abbreviations: CI indicates confidence interval; OR, odds ratio.

* p:0.003.

** p:0.006.

Comorbid conditions and hospital center maintained their independent association. We introduced a robust estimation of variance to our multivariate logistic models to control the possible correlation among different observations belonging to the same centre. The χ^2^ goodness-of-fit analysis demonstrated the adequacy of the model (p = 0.2).

## Discussion

The main findings of our study are the following: 1. Lack of compliance with processes of care depends on general health status measured using the Charlson index but not on age or clinical signs. 2. Prescription of a nonadherent antibiotic is higher in patients with a high Charlson score, confusion and tachycardia. 3. Delayed first antibiotic dose (>6 hours) was lower in patients with tachycardia. 4. Lack of initial oxygen assessment was associated with fever, whereas tachypnea, age (>65) and COPD were protective factors. 5. Compliance with both antibiotic adherence and timing <6 hours was lower in patients with poorer health status, measured by Charlson score, and confusion.

There is wide variation in performance and even adherence to guidelines varies between hospitals and physicians. Barriers to compliance with adherence may be due to different reasons [15] and educational programs have been implemented to improve quality of care. In order to improve quality of care, it is essential to identify risk factors and/or predictors of underuse of or noncompliance with guidelines. Factors depending on hospital characteristics may be managed using local measures and probably are not applicable to other hospitals. However, focusing on initial patient characteristics and/or signs may be more feasible for a faster dissemination of improvement [2]. In fact, knowledge of factors relating to patient comorbidities and health status may be more useful for increasing awareness among physicians regardless of specialty and area.

In our study, the quality measures selected were antibiotic timing, oxygen assessment and antibiotic adherence, i.e., processes that reflect care at the time of diagnosis and admission and clearly depend on physicians and the staff in charge**.** In prior studies, it has been confirmed that compliance with more than one process of care improved survival in hospitalized CAP, even when associated with severe sepsis [5], [6], [21].

Antibiotic adherence to guidelines is the most studied process of care and the one most consistently linked to improved outcome [3], [5], [6], [21]–[24]. Barriers to selecting recommended antibiotics may depend on specialty [24], [25], physician characteristics [26] and hospital [7]. That is why focusing on patients makes prevention of noncompliance more feasible. Our findings may alert about specific populations not adequately addressed by guidelines and, more importantly, patients at risk of receiving non-recommended antibiotics such as those with a high Charlson score. It is plausible that patients with a poor functional heath status measured by the Charlson score have been managed more as disabled elderly or health care associated pneumonia (HCAP) than as CAP. In fact, the concept of HCAP is making it clear that diagnostic algorithms in patients with several comorbidities or very disabled patients need a different etiological approach [27].

Timing until the first antibiotic dose is the subject of debate [9], [11], [28]. Nevertheless, there is agreement that greater severity requires treatment to be provided sooner [5], [29]. In our study, patients with tachycardia were treated earlier, suggesting that this vital sign is still better recognized than respiratory rate. Interestingly, our findings were adjusted by hospital because that measure is related to several characteristics, such as hospital type and staff-bed ratio, as previously reported [30]. Fine et al [2] found higher performance of antibiotic timing and blood culture collection in hospitals with a nurse-bed ratio higher than 1.25.

Early initial oxygen assessment in severe pneumonia improves survival [12], is easy to perform with saturation and is highly recommended. In our study, we found that adults under 65 years of age were less often evaluated, [31] while those with COPD were, as expected, more frequently assessed. That is, we have a better recognition of hypoxemia concerns in patients with increased respiratory rate, in the elderly and in COPD. On the other hand, we still underestimate the risk in young adults and we miss that evaluation in those with confusion. This is of concern because confusion is a severity factor related to mortality.

Some investigators have demonstrated improvements in survival based on implementation of combinations of processes of care [5], [32]; thus, we investigated the risk factors relating to lack of compliance with combinations of two or three processes of care. As expected, similar findings were associated with receiving care that did not comply with two or three processes of care in CAP. Interestingly, a deficient health status, identified by a high Charlson score, rather than age, also highlights the fact that disabled patients are diagnosed later and treated with different antibiotic regimens.

### 

#### Limitations

Our study did not evaluate differences between hospitals, factors related to organization, or night/weekend admission, or differences among doctor characteristics that might also influence compliance with processes of care because we focused on patient characteristics. The strength is that our findings provide information that is useful for improving adherence (an area for improvement), with the advantage that it focuses on patient health status and initial presentation. This approach can be easily applied to physicians from any hospital type, thereby making processes of care amenable to quality improvement.

In summary, our findings highlight underuse of the main recommended processes of care in hospitalized CAP patients. We have found that a poorer health status along with confusion, and not age, was associated with lower compliance with quality of care in choice and administration time of antibiotics. These results should contribute to improving awareness of clinicians and targeting specific disabled patients who receive poorer quality of care and who are easily identifiable on initial evaluation, when our decisions are better able to affect outcome. Our findings may be useful for quality improvement aimed at better recognition of lack of compliance based on patient comorbid condition and clinical signs.
